# Schizophrenia management: Systematic review of current medications and Phase-3 agents (2008–2024)

**DOI:** 10.1016/j.nsa.2025.105507

**Published:** 2025-02-06

**Authors:** Salma Abdelmoteleb, Jayant Totlani, Salma Ramadan, Mohamed Salem, Ashley Meyer, Tiffany Chang, Madeline Ewing, Luiza Freire, Nathalie Murphy, Sabrina Renteria, Romana Dymkoski, Omer Liran, Rebecca Hedrick, Itai Danovitch, Robert N. Pechnick, Waguih William IsHak

**Affiliations:** a Cedars-Sinai Health System, Los Angeles, CA, USA; b Virginia Commonwealth University Health System, Richmond, VA, USA; c Brody School of Medicine at East Carolina University Greenville, NC, USA; d University of California Irvine, School of Medicine, Irvine, CA, USA; e Faculdade Pernambucana de Saude, Recife, PE, Brazil; f Western University of Health Sciences, Pomona, CA, USA; g David Geffen School of Medicine at UCLA, Los Angeles, CA, USA

**Keywords:** Psychiatric medications, Schizophrenia, Psychosis, Psychiatric disorders

## Abstract

This systematic review evaluates psychiatric medications for schizophrenia approved between 2008 and 2024, considering regulatory practices and approvals across multiple regions, including the United States, Europe, and Asian countries. It details the mechanism of action, indications, efficacy, dosing, and adverse effects of each medication. The methodology involved a literature search of the PubMed database for studies published from 2008 to December 31, 2024 on FDA-approved psychiatric medications and Phase 3 pipeline medications, along with additional medications approved in Europe using the keywords “schizophrenia” OR “psychosis” AND “psychopharm∗” OR “medic∗” OR “pharm∗.” An independent assessment was conducted, followed by a consensus on eligible studies for inclusion in the systematic review. From 2008 to December 31, 2024, the FDA approved 29 medications for schizophrenia including 13 long-acting injectables (LAI), in addition to that there are additional three other medications that are available in Europe but not in the United States. Additionally, 8 pipeline medications are currently in Phase-3 clinical trials including one LAI. Each medication is analyzed, and its mechanisms of action, indications, dosing, efficacy, and adverse effects are described. The 13 approved LAIs and the one LAI in the pipeline are detailed in a separate manuscript. This review highlights a significant increase in approved medications for the treatment of schizophrenia, including long-acting injectable formulations that enhance the range of existing oral therapies. Furthermore, new treatments for medication-related movement disorders have been introduced. Innovative developments in Phase-3 trials for schizophrenia medications, including new mechanisms of action and administration routes, promise to transform treatment strategies and enhance patient outcomes.

## Abbreviations

5-HT: 5-Hydroxytryptaminem
AIMS: Abnormal Involuntary Movements Scale
BPRS: Brief Psychiatric Rating Scale
CGI: Clinical Global Impression
D2: Dopamine D2 receptor
ER: Extended-Release
GlyT1: Glycine Transporter-1
HD: Huntington's Disease
IEM: Ingestible Event Maker
MDD: Major Depressive Disorder
NMDA: N-Methyl-D-Aspartate
PANSS: Positive and Negative Syndrome Scale
PEC: in Positive and Negative Syndrome Scale-Excited Component
TD: Tardive Dyskinesia
VMAT-2: Vesicular Monoamine Transporter 2

## 1 Introduction

Schizophrenia is a chronic and debilitating disorder that affects one's perception of reality. It is characterized by disruptions in thought processes, perceptions, emotional responsiveness, and social interactions (National Institute of Mental Health). Symptoms typically manifest through a combination of psychotic features, which may include hallucinations, delusions, and disorganized thinking, as well as negative symptoms such as reduced emotional expression and motivation, difficulties in social relationships, and cognitive impairments (Hollis et al., 2011; McGrath et al., 2008). The onset of schizophrenia usually occurs in late adolescence to early adulthood and often appears earlier in males than females. If left untreated, the disorder can be severely disabling and persistent (Hollis et al., 2011; McGrath et al., 2008) (see Table 1).

**Table 1:** Approved medications for Schizophrenia 2008–December 31, 2024.

N	APPROVED MEDICATIONS/Approval Year		
29	Generic Name	Trade Name	Approval Date
	1. Aripiprazole^a^	Abilify Asimtufii	2023
	2. Aripiprazole^a^	Abilify Maintena	2013
	3. Aripiprazole Lauroxil a	Aristada	2015
	4. Aripiprazole^a^	Aristada Initio	2018
	5. Aripiprazole ODT	Abilify Discmelt	2015
	6. Aripiprazole oral film	Opipza	2024
	7. Aripiprazole tablets with sensor	Abilify MyCite	2017
	8. Asenapine SL	Saphris	2009∗∗
	9. Asenapine Transdermal	Secuado	2021
	10. Brexpiprazole	Rexulti	2015∗∗
	11. Cariprazine	Vraylar	2015∗∗
	12. Clozapine Suspension	Versacloz	2013
	13. Dexmedetomidine SL	Igalmi	2022
	14. Iloperidone	Fanapt	2009∗∗
	15. Loxapine inhalation	Adasuve	2012
	16. Lumateperone Tosylate	Caplyta	2019∗∗
	17. Lurasidone	Latuda	2010∗∗
	18. Olanzapine Pamoate^a^	Zyprexa Relprevv	2009
	19. Olanzapine + Samidorphan	Lybalvi	2021
	20. Paliperidone Extended release	Invega	2009
	21. Paliperidone Palmitate^a^	Invega Sustenna	2009
	22. Paliperidone Palmitate^a^	Erzofri	2024
	23. Paliperidone Palmitate^a^	Invega Trinza	2019
	24. Paliperidone Palmitate^a^	Invega Hafyera	2021
	25. Risperidone SC^a^	Perseris	2019
	26. Risperidone SC^a^	Uzedy	2023
	27. Risperidone IM^a^	Rykindo	2023
	28. Risperidone IM^a^	Risvan	2024
	29. Xanomeline plus trospium chloride	Cobenfy	2024∗∗

a Long-acting injectable (LAI) antipsychotics which are fully detailed by the authors in a separate manuscript. ∗∗ New Drug Application approved by the FDA (medication has not been previously approved for another indication).

In the United States, the prevalence of schizophrenia is estimated to be between 0.25% and 0.64%, with similar rates internationally ranging from 0.33% to 0.75%. Despite its relatively low prevalence, schizophrenia poses a major public health challenge due to its profound health, social, and economic impacts (Saha et al., 2005; Desai et al., 2013; Kessler et al., 2005; Moreno-Küstner et al., 2018; Wu et al., 2006). Approximately 4.9% of individuals with schizophrenia die by suicide, a rate significantly higher than that of the general population (Palmer et al., 2005). It ranks among the top 15 leading causes of disability worldwide and is linked with an increased risk of premature mortality, including an average potential life loss of 28.5 years (Olfson et al., 2015). The disorder also correlates with a higher incidence of comorbidities, including heart disease, liver disease, and diabetes, contributing to an elevated mortality rate (Laursen et al., 2014; Schoenbaum et al., 2017; Simon et al., 2018; Tsai and Rosenheck, 2013).

Schizophrenia also incurs substantial economic burdens through both direct costs, such as healthcare expenses, and indirect costs, including lost productivity, criminal justice involvement, and social service demands. These costs are disproportionately high relative to other chronic mental and physical health conditions (Desai et al., 2013).

Since the advent of chlorpromazine in the 1950s, medications have become the first line of treatment for both positive and negative symptoms of schizophrenia, especially when prescribed with careful consideration of risk factors and adverse effects. The field has continued to expand the formulary, diversify routes of administration, and minimize side effects, to enhance patient care and quality of life.

Over the past fifteen years, there has been significant progress in the development of new medications for schizophrenia. This systematic review evaluates psychiatric medications for schizophrenia approved between 2008 and December 31, 2024, considering regulatory practices and approvals across multiple regions, including the United States, and Europe. It details the mechanism of action, indications, evidence for efficacy, dosing, and adverse effects of these medications. The goal is to provide prescribers with comprehensive information about the approved psychiatric medications in the past 15 years and those in Phase 3 development, thereby enhancing clinicians’ familiarity with the latest medications for schizophrenia.

## 2 Methods

### 2.1 Design and registration

This systematic review investigates advancements in the approved psychiatric medications and Phase 3 pipelines drugs, for treatment of schizophrenia and psychosis, across multiple regions, including the FDA approved medications in the United States and additional medications approved in Europe. The methodology adheres to established guidelines for systematic reviews, following the Preferred Reporting Items for Systematic Reviews and Meta-Analyses (PRISMA) framework.

### 2.2 Search strategy

A comprehensive search was conducted in PubMed and Web of Science databases for studies published between 2008 and December 31, 2024. The search employed Boolean operators to combine terms, including:

• “schizophrenia” OR “psychosis” AND
• “psychopharm∗” OR “medic∗” OR “pharm∗”.

Additionally, the reference lists of selected studies were manually screened for relevant articles. Search terms were refined iteratively to ensure coverage of all relevant literature.

### 2.3 Selection criteria

Studies were eligible for inclusion if they met the following criteria:

• Peer-reviewed publications in English.
• Clinical trials, systematic reviews, or observational studies on FDA-approved psychiatric medications or Phase 3 pipeline drugs.
• Focus on schizophrenia or psychosis.

Exclusion criteria were:

• Studies not involving schizophrenia or psychosis.
• Articles lacking original data or employing outdated methodologies.
• Publications not meeting systematic review standards.

### 2.4 Data extraction

Key data were extracted from eligible studies, includin study design, patient population, and primary outcomes**.** An independent initial assessment of all identified studies was performed by two reviewers. Following this, a consensus process was conducted to finalize study selection, ensuring methodological rigor and relevance (IsHak et al., 2024; IsHak et al., 2024). Any disagreements were resolved by consultation with a third expert.

## 3 Results

From 2008 to December 31, 2024, the FDA approved 29 new medications for schizophrenia including 13 LAIs, which are detailed in a separate manuscript on LAIs (Meyer et al., 2024), in addition to that there are additional three other medications that are available in Europe but not in the united states. Additionally, 8 pipeline medications for schizophrenia are currently in Phase 3 clinical trials, including one LAI, which is detailed in a separate manuscript on LAIs (Meyer et al., 2024).

For each approved medication, this review provides the medication class, mechanism of action, indications for labeled and off-label uses, evidence supporting efficacy, and practical implementation issues such as cost, special procedures, and potential restrictions. Additionally, the reported adverse effects are discussed (see Table 2).

**Table 2:** Pipeline medications for schizophrenia in Phase 3 as of December 31, 2024.

N	PHASE-3 MEDICATION		
8	Generic Name	Brand Names	Phase 3 Start Date
	1. Brilaroxazine	Oxaripiprazole, RP-5063	January 2022
	2. Iclepertin	BI-425809	June 2021
	3. Raloxifene (adjunctive therapy)	Evista	March 2011
	4. Roluperidone	MIN-101, CYR-101, MT-210	December 2017
	5. Sodium Benzoate	NaBen	March 2021
	6. Ulotaront	SEP-363856	October 2019
	7. Vortioxetine	Trintellix	January 2022
	8. Olanzapine SC^a^	TV-44749	January 2023

a Long-acting injectable (LAI) antipsychotic which is fully detailed by the authors in a separate manuscript.

**Table 3:** Summary descriptions of approved non-LAI medications from 2008 to December 31, 2024.

Name, Year approved	Mechanism of Action	Route and Dose	Notes to clinicians including effects on sedation, weight/lipids, extrapyramidal tract, prolactin, and QTc
1. Aripiprazole ODT (generic Abilify Discmelt), 2015	D2 receptor partial agonist, 5-HT1A receptor partial agonist, and 5-HT2A receptor antagonist	Orally disintegrating tablet 15–20 mg daily	Akathisia.
2. Aripiprazole oral film (Opipza), 2024	D2 receptor partial agonist, 5-HT1A receptor partial agonist, and 5-HT2A receptor antagonist	Oral 2–30 mg daily	Akathisia.
3. Aripiprazole tablets with sensor (Abilify MyCite)	D2 receptor partial agonist, 5-HT1A receptor partial agonist, and 5-HT2A receptor antagonist	Oral 15–20 mg	Akathisia.
4. Asenapine SL (Saphris), 2009	Antagonist at D2 and 5-HT2A receptors	Sublingual 5 mg–10 mg twice daily	Akathisia, somnolence, and extrapyramidal side effects.
5. Asenapine transdermal (Secuado), 2021	Antagonist at D2 and 5-HT2A receptors	Transdermal 3.8 mg–7.6 mg/24 h	Weight gain (>7% from baseline): 5.5% (5–10 mg bid) vs. 0.4% PBO, sedation: 23% (5–10 mg bid) vs. 5% PBO, Moderate effect on QTc, in addition to tiredness, dizziness, somnolence, and insomnia.
6. Brexpiprazole (Rexulti), 2015	Partial agonist at D2 and 5-HT1A receptors	Oral 2–4 mg daily	Akathisia, somnolence, and weight gain.
7. Cariprazine (Vraylar), 2015	Partial agonist at D2, D3, and 5-HT1A receptors, and antagonist at 5-HT2A receptors	Oral 1.5 mg–4.5 mg daily	Akathisia, insomnia.
8. Clozapine Suspension (Versacloz), 2013	Antagonist at 5-HT2A, 5-HT2C, D1, and D4 receptors	Oral 12.5–900 mg daily	QT prolongation, sedation, weight gain, dyslipidemia, and risk of severe neutropenia. Only available through REMS program.
9. Dexmedetomidine sublingual (Igalmi), 2022	Alpha-2 adrenergic receptor agonist	Sublingual 120 μg-180 μg daily	Somnolence, hypotension, bradycardia, withdrawal symptoms, arrhythmia and QTc prolongation.
10. Iloperidone (Fanapt), 2009	D2 and 5-HT2A receptor antagonist	Oral 6–12 mg twice daily	QTc prolongation, weight gain, and sedation.
11. Loxapine (Adasuve), 2012	Antagonist at D1, D2 and 5-HT2 receptors	Inhalation powder 10 mg, once per day maximum.	Sedation (12%) Only available at approved facilities under FDA-required REMS program (black box warning for bronchospasm).
12. Lumateperone Tosylate (Caplyta), 2019	5-HT2A and D2 receptor antagonist	Oral 42 mg daily	Sedation (24%). Low frequency of EPS, weight gain, prolactin changes, and QTc prolongation.
13. Lurasidone (Latuda), 2010	D2 and 5-HT2A receptor antagonist	Oral 40–80 mg daily	Weight gain, and somnolence.
14. Olanzapine and Samidorphan (Lybalvi), 2021	Samidorphan is an opioid antagonist (to prevent olanzapine's effect on weight and metabolism)	Oral 5 mg/10 mg–20 mg/10 mg	Insomnia, increased ALT and increased CPK.
15. Paliperidone Extended release (Invega), 2009	D2 and 5HT2A receptor antagonist; also, antagonist at alpha-1 and alpha-2 and H1 histamine receptors	Oral 3–12 mg daily	Akathisia, insomnia, EPS.
16. Xanomeline + Trospium chloride (Cobenfy), 2024	Muscarinic M1/M4 agonist (xanomeline) and peripherally acting muscarinic antagonist (trospium chloride)	Oral 50mg/20 mg - 125 mg/30 mg twice daily	Nausea, dyspepsia, constipation, vomiting, hypertension, abdominal pain, diarrhea, tachycardia, dizziness, and gastrointestinal reflux disease.

**Table 4:** Summary descriptions of phase- 3 medications for non-LAI Schizophrenia as of December 31, 2024.

Name	Mechanism of action	Indications being tested in Phase 3	Route and dose	Notes to clinicians (including effects on sedation, weight/lipids, extrapyramidal tract, prolactin, and QTc)
1. Brilaroxazine (Oxaripiprazole, RP-5063)	Partial agonist at dopamine D2, D3, D4, partial agonist at serotonin 5-HT1A, 5-HT2A, 5-HT2B receptors, and antagonist at serotonin 5-HT6 and 5-HT7 receptors	Cognitive dysfunction and psychotic symptoms in schizophrenia	Oral, 5–100 mg daily	Somnolence and akathisia were detected, but no reported changes in weight, lipid or prolactin levels, or EKG.
2. Iclepertin (BI-425809)	Glycine transporter 1-inhibitor	Cognitive dysfunction in schizophrenia	Oral, 2–25 mg daily	Did not demonstrate improvements on the Schizophrenia Cognition Rating Scale (SCoRS), which is a requirement for approval.
3. Raloxifene	Estrogen receptor modulator	Adjunctive therapy for psychotic and cognitive symptoms for postmenopausal women with schizophrenia	Oral, 120 mg daily	Weight gain was detected.
4. Roluperidone (MIN-101)	Serotonin 5-HTA and alpha 1-a adrenergic receptor antagonist	Negative symptoms in schizophrenia	Oral, 32–64 mg daily	No extrapyramidal side effects, no weight or metabolic effects, and no changes in prolactin levels.
5. Sodium Benzoate (NaBen)	D-amino acid oxidase (DAAO) inhibitor: enhances NMDAR function	Adjunctive treatment for schizophrenia and clozapine-resistant schizophrenia	Oral, 500–1000 mg twice a day	Dose-dependent efficacy increase and favorable safety profile. Detected extra-pyramidal in the sodium benzoate group compared to placebo.
6. Ulotaront (SEP-363856)	Trace-amine associated receptor 1 (TAAR-1) agonist	Psychotic symptoms in schizophrenia	Oral, 25–75 mg daily	No extrapyramidal side effects, low weight and metabolic effects, and no changes in prolactin levels.
7. Vortioxetine (Trintellix)	5-HT multimodal agent: agonist at 5-HT1A, antagonist at 5-HT3, 5-HT7 and 5-HT1D, partial agonism at 5-HT1B, and inhibitor of the 5-HT transporter (SERT)	Cognitive functioning and negative symptoms in early-stage schizophrenia	Oral, 5–20 mg daily	Common: Nausea, constipation, vomiting. Risk of activation of mania, bleeding, fragility fractures, hyponatremia, serotonin syndrome, sexual dysfunction, suicidal thinking/behavior, withdrawal syndrome. Might require dose with CYP2D6 inducers or inhibitors.

## 4 Detailed descriptions of FDA approved medications for schizophrenia 2008–2024 (summarized in Table 3)

The approved schizophrenia medications from 2008-2024 are detailed below, except for LAIs, which are covered in another paper (Meyer et al., 2024).

### 4.1 Aripiprazole orally disintegrating tablets (ODT) (Abilify Discmelt)

#### 4.1.1 Overview

Generic Abilify Discmelt is an orally disintegrating formulation of the antipsychotic aripiprazole. Aripiprazole functions as a partial agonist at dopamine D2 and serotonin 5-hydroxytryptamine (5-HT)-1A receptors and as an antagonist at 5-HT2A receptors. Initially approved for schizophrenia and acute treatment of manic and mixed episodes in Bipolar I Disorder in adults, the original Abilify Discmelt was discontinued in 2009 for reasons unrelated to safety or efficacy. The generic form received FDA approval in 2015.

Indications:

• Schizophrenia in adults.
• Acute treatment of manic and mixed episodes in bipolar I disorder in adults (generic form in 2015).

#### 4.1.2 Dose and route

Orally disintegrating tablet 15–20 mg daily.

#### 4.1.3 Evidence

Oral aripiprazole has demonstrated efficacy in managing acute schizophrenia symptoms and preventing relapses. Additionally, it is approved in the United States for bipolar mania, as an adjunctive treatment in major depressive disorder, irritability in autism, and Tourette's disorder, with similar indications in Europe. Short-term studies showed that 10–30 mg/day dosages improved psychometric scores within the first week. A 26-week study indicated that aripiprazole significantly reduced relapse rates in stable schizophrenia patients compared to placebo (Pigott et al., 2003).

#### 4.1.4 Practical implementation issues

The medication remains relatively expensive despite its generic status.

#### 4.1.5 Adverse effects

• Akathisia.

### 4.2 Aripiprazole oral film (Opipza)

**Overview:** Opipza is a novel aripiprazole formulation in oral film, designed to simplify administration. The film dissolves on the tongue and is swallowed without water, making it easier for patients with swallowing difficulties. It can be taken with or without food. In addition to treating schizophrenia, Opipza is approved for adjunctive treatment of depression, and irritability associated with autism spectrum disorder, and Tourette's disorder. Available in 2 mg, 5 mg, and 10 mg forms.

#### 4.2.1 Indications

• Treatment of schizophrenia in patients aged 13 years and older
• Adjunctive treatment of major depressive disorder (MDD) in adults
• Irritability associated with autistic disorder in pediatric patients aged 6 years and older.
• Treatment of Tourette's disorder in pediatric patients aged 6 years and older.

#### 4.2.2 Dose and route

Oral 2–30 mg once daily.

#### 4.2.3 Evidence

In a 6-week, placebo-controlled trial involving 302 pediatric patients (ages 13 to 17) with schizophrenia, both 10 mg/day and 30 mg/day doses of aripiprazole significantly improved PANSS total scores compared to placebo. However, the 30 mg/day dose did not provide additional benefit over the 10 mg/day dose (Findling et al., 2008).

#### 4.2.4 Practical implementation issues

Approved in July 2024, Opipza may encounter initial availability and insurance coverage challenges as it enters the market.

#### 4.2.5 Adverse effects

• Akathisia

### 4.3 Aripiprazole tablets with sensor (Abilify Mycite)

#### 4.3.1 Overview

Abilify MyCite integrates a sensor with the antipsychotic aripiprazole. It is approved for schizophrenia, acute and maintenance treatment of bipolar I disorder, and as an adjunct in major depressive disorder (MDD). This system monitors medication adherence using an ingestible event marker (IEM) sensor embedded in the aripiprazole tablet, complemented by a wearable sensor (MyCite Patch), a smartphone app (MyCite App), and a web portal for caregivers and healthcare professionals.

#### 4.3.2 Indications

• Treatment of adults with schizophrenia (2017).
• Treatment of bipolar I disorder (2017): Acute treatment of manic and mixed episodes (monotherapy and adjunct to lithium or valproate), maintenance treatment (monotherapy and adjunct to lithium or valproate), and adjunctive treatment for MDD.

#### 4.3.3 Dose and route

Oral 15–20 mg.

#### 4.3.4 Evidence

Sensor-enabled aripiprazole improved Positive and Negative Syndrome Scale (PANSS) scores by 12.2% over 12 months and reduced hospitalizations by 28.2% (Chopra et al., 2023). Higher engagement with the Abilify MyCite system was associated with significant improvements in clinical scores for schizophrenia (Cochran et al., 2022).

#### 4.3.5 Practical implementation issues

Despite being available in generic form, the medication remains costly.

#### 4.3.6 Adverse effects

• Akathisia, restlessness, tremors, and extrapyramidal symptoms (EPS).

### 4.4 Asenapine SL (Saphris)

#### 4.4.1 Overview

Asenapine is a sublingual dopamine D2 and 5-HT2A receptor antagonist approved in 2009 for schizophrenia. It received further approval in 2015 to treat bipolar disorder.

#### 4.4.2 Indications

• Schizophrenia in adults (2009).
• Bipolar I disorder: Acute monotherapy treatment of manic or mixed episodes in adults (2009) and pediatric patients aged 10–17 years (2015), adjunctive treatment to lithium or valproate in adults (2009), and maintenance monotherapy treatment in adults (2017).

#### 4.4.3 Dose and route

Sublingual 5 mg–10 mg twice daily.

#### 4.4.4 Evidence

Both short-term and long-term randomized controlled studies demonstrated that 5 mg of asenapine twice daily is more effective than a placebo in managing acute schizophrenia symptoms over eight weeks. Adjusted doses, typically 10 mg twice daily, effectively sustain treatment response over 26 weeks (Orr et al., 2017).

#### 4.4.5 Practical implementation issues

Asenapine is the only approved sublingual antipsychotic (Balaraman and Gandhi, 2010).

#### 4.4.6 Adverse effects

• Tiredness, dizziness, somnolence, insomnia, and mild QT prolongation.
• Somnolence (23% for 5–10 mg bid) vs. placebo (5%).
• Weight gain (>7% from baseline): 5.5% (5–10 mg bid) vs. 0.4% (placebo).

### 4.5 Asenapine transdermal (Secuado)

#### 4.5.1 Overview

Approved in 2021, Asenapine transdermal (Secuado) is the first and only FDA-approved antipsychotic patch for adults with schizophrenia. It acts as a dopamine D2 and 5-HT2A receptor antagonist.

Indications:

• Schizophrenia (2021).

#### 4.5.2 Dose and route

Transdermal 3.8 mg–7.6 mg/24 h**.**

#### 4.5.3 Evidence

The clinical efficacy was established through a 6-week randomized, fixed-dose, placebo-controlled Phase 3 trial involving 617 subjects. Significant improvements in PANSS and clinical global impression – severity scale (CGI-S) scores were observed (Citrome et al., 2020).

#### 4.5.4 Practical implementation issues

This transdermal formulation is costlier than the sublingual form. Application sites include the upper arm, upper back, abdomen, or hip, with daily site rotation required. Metabolized by CYP1A2; caution is advised when used with fluvoxamine. It is contraindicated in Child-Pugh class C hepatic impairment and cases of hypersensitivity to asenapine. It is not approved for dementia-related psychosis (Citrome et al., 2020).

#### 4.5.5 Adverse effects

• Somnolence, dizziness, headache, insomnia, and fatigue.
• Cerebrovascular events, including stroke, neuroleptic malignant syndrome, tardive dyskinesia (TD), and metabolic changes.

### 4.6 Brexpiprazole (Rexulti)

#### 4.6.1 Overview

Brexpiprazole is an orally administered medication that acts as a partial agonist at serotonin 5-HT1A and dopamine D2 receptors and antagonist at serotonin 5-HT2A receptors. It has been approved for treating schizophrenia, as an adjunctive treatment in major depressive disorder, and for managing agitation in dementia patients.

#### 4.6.2 Indications

• Schizophrenia (2015).
• Adjunct to antidepressants in MDD (2015).
• Agitation in dementia (2023) as a standing medication.
• It is also used off-label for bipolar depression, with phase 3 studies currently ongoing.

#### 4.6.3 Dose and route

Oral 2–4 mg daily.

#### 4.6.4 Evidence

Brexpiprazole demonstrated significant improvements in PANSS scores compared to aripiprazole. Clinical studies showed efficacy in reducing agitation and psychotic symptoms (Citrome et al., 2016).

#### 4.6.5 Practical implementation issues

Brexpiprazole has a similar pharmacodynamic profile to aripiprazole but with fewer cognitive adverse effects (Das et al., 2016).

#### 4.6.6 Adverse effects

• Akathisia, somnolence, headache, weight gain, and tremors (Bruijnzeel and Tandon, 2016).

### 4.7 Cariprazine (Vraylar)

#### 4.7.1 Overview

Cariprazine is an orally administered medication with partial agonist activity at central dopamine D2 and D3, serotonin 5-HT1A receptors, and antagonist activity at serotonin 5-HT2A, 5-HT2 alpha, and 5-HT2 beta receptors. It exhibits significantly less antagonism at 5-HT2C, 5-HT7, and Histamine-1 receptors (Scarff, 2016). It also acts on NMDA glutamatergic receptors in the prefrontal cortex (Titulaer et al., 2022).

#### 4.7.2 Indications

• Schizophrenia (2015).
• Depressive episodes associated with bipolar I disorder (2017).
• Acute treatment of manic or mixed episodes in bipolar I disorder (2019).
• Adjunctive therapy for major depressive disorder (2022).

#### 4.7.3 Dose and route

Oral 1.5 mg–4.5 mg daily.

#### 4.7.4 Evidence

Cariprazine was extremely effective in treating patients with schizophrenia symptoms (n = 732) with all doses (PANSS: [1.5 mg, −17.3; 3 mg, −18.7; 4.5 mg, −20.2, p < 0.001]; CGI-S [1.5 mg, −0.9; 3 mg, −1.1; 4.5 mg, −1.2]) (Durgam et al., 2014). Another phase-3 trial also observed significant improvement in schizophrenic symptoms with the administration of cariprazine (3–6 mg/day, or 6–9 mg/day). PANSS total scores were used to evaluate efficacy (3–6 mg, −6.8; 6–9 mg, −9.9; p < 0.001) (Kane et al., 2015).

#### 4.7.5 Practical implementation issues

Delayed adverse reactions may occur due to cariprazine's long half-life and metabolites (Kiss et al., 2019).

#### 4.7.6 Adverse effects

• Akathisia, nausea, constipation, and decreased prolactin levels.
• Not recommended for patients with dementia-derived psychosis due to cerebrovascular complications (Scarff, 2016).

### 4.8 Clozapine suspension (Versacloz)

#### 4.8.1 Overview

Clozapine, primarily used for treatment-resistant schizophrenia and to reduce suicidal behavior in schizophrenia or schizoaffective disorder is the subject of FDA guidance documents that recommend bioequivalence studies for its generic versions (DailyMed, 2025; U.S. Food and Drug Administration).

#### 4.8.2 Indications

1. **Treatment-resistant schizophrenia**: Clozapine is indicated for patients who have not responded adequately to standard antipsychotic treatments.
2. **Reduction of suicidal behavior**: It is used to decrease the risk of recurrent suicidal behavior in patients with schizophrenia or schizoaffective disorder.

#### 4.8.3 Dose and route

Oral 12.5–900 mg daily.

#### 4.8.4 Evidence

##### 4.8.4.1 Treatment-resistant schizophrenia

The efficacy of clozapine for treatment-resistant schizophrenia was established in a 6-week, randomized, double-blind, active-controlled study comparing clozapine to chlorpromazine. This pivotal study demonstrated clozapine's superior efficacy in patients who had failed to respond to other antipsychotics (Kane et al., 1988). Additional studies have demonstrated that clozapine significantly reduces positive symptoms, negative symptoms, and overall psychopathology compared to other antipsychotics in patients who are unresponsive to standard treatments.

##### 4.8.4.2 Reduction of suicidal behavior

The InterSePT™ (International Suicide Prevention Trial) provided substantial evidence supporting the use of clozapine in reducing suicidal behavior. This two-year study involved patients with schizophrenia or schizoaffective disorder at high risk for suicide. The results showed a significant reduction in suicidal behaviors in patients treated with clozapine compared to those treated with olanzapine (Alphs et al., 2004).

#### 4.8.5 Practical implementation issues

Clozapine administration starts at 12.5 mg once or twice daily, gradually increasing to a target of 300–450 mg/day, with ongoing treatment continuing beyond the acute phase. Discontinuation should be tapered over 1–2 weeks unless severe neutropenia occurs. Monitoring requirements include baseline and ongoing absolute neutrophil count (ANC) checks, with weekly tests for the first six months, bi-weekly for the next six months, and monthly thereafter. Dosage adjustments are necessary when taken with CYP1A2, CYP2D6, or CYP3A4 inhibitors or inducers.

Participation in the REMS program is mandatory. Healthcare providers, patients, and pharmacies must ensure compliance with ANC monitoring and reporting. Clozapine has boxed warnings for severe neutropenia, orthostatic hypotension, bradycardia, syncope, seizures, myocarditis, cardiomyopathy, and increased mortality in elderly patients with dementia-related psychosis (Haidary and Padhy, 2024).**)**

#### 4.8.6 Adverse effects

Clozapine has several severe adverse effects, including:

• **Severe neutropenia**: A significant reduction in neutrophils, increasing infection risk.
• **Orthostatic hypotension, bradycardia, and syncope**: These can occur, especially during initial dose titration.
• **Seizures**: Dose-related and requires careful monitoring.
• **Myocarditis and cardiomyopathy**: Can be fatal; requires discontinuation if suspected.
• **Increased mortality in elderly with dementia-related psychosis**: Higher death rates in this population.
• **Other effects**: Gastrointestinal hypomotility, eosinophilia, QT prolongation, metabolic changes (hyperglycemia, dyslipidemia, weight gain), neuroleptic malignant syndrome, hepatotoxicity, fever, pulmonary embolism, anticholinergic toxicity, cognitive and motor performance interference, tardive dyskinesia, cerebrovascular adverse reactions, and cholinergic rebound upon abrupt discontinuation (Haidary and Padhy, 2024).

### 4.9 Dexmedetomidine sublingual (Igalmi)

#### 4.9.1 Overview

Dexmedetomidine SL is an orally administered sublingual film that targets and activates presynaptic adrenergic receptors as an alpha-2 adrenergic receptor agonist. While dexmedetomidine was granted initial approval in 1999 for intravenous use, the sublingual form was approved in 2022 for treating agitation in patients with schizophrenia and bipolar disorder (types I and II) (Stewart). Studies have demonstrated that it effectively and significantly reduces acute agitation within only 20 min of administration (BioXcel Therapeutics and Inc, 2022).

#### 4.9.2 Indications

• Treatment of agitation in adults with schizophrenia and bipolar disorder (I and II) (2022).

#### 4.9.3 Dose and route

Sublingual 120–180 μg daily.

#### 4.9.4 Evidence

The SERENITY I and II trials demonstrated significant reductions in Positive and Negative Syndrome Scale-Excited Component (PEC) scores at 20 min post-administration for both 180 μg and 120 μg doses (Citrome et al., 2022).

#### 4.9.5 Practical implementation issues

Dosage reduction is advised for geriatric patients and those with arrhythmias. Intravenous dexmedetomidine 24 h post-administration may lead to tachyphylaxis.

#### 4.9.6 Adverse effects

• Somnolence, hypotension, bradycardia, arrhythmia, and QT prolongation.
• Withdrawal symptoms (nausea, vomiting, agitation) may occur within 48 h of discontinuation.

### 4.10 Iloperidone (Fanapt)

#### 4.10.1 Overview

Iloperidone (Fanapt) is an atypical antipsychotic approved for treating schizophrenia and acute manic or mixed episodes of bipolar I disorder in adults (Jeun et al., 2024).

#### 4.10.2 Indications

Iloperidone is indicated for the treatment of schizophrenia in adults and the acute treatment of manic or mixed episodes associated with bipolar I disorder in adults.

#### 4.10.3 Dose and route

Oral 6–12 mg twice daily.

#### 4.10.4 Evidence

##### 4.10.4.1 Schizophrenia

Randomized controlled trials have shown that iloperidone significantly improves symptoms of schizophrenia compared to placebo in adults. These trials typically measure symptom improvement using standardized assessment tools such as the PANSS or the Brief Psychiatric Rating Scale (BPRS). Patients treated with iloperidone have shown statistically significant reductions in psychotic symptoms, including positive symptoms like hallucinations and delusions, as well as negative symptoms such as social withdrawal and blunted affect (Montes and Rey, 2009).

##### 4.10.4.2 Bipolar I disorder

Clinical trials have shown that iloperidone is effective in managing acute manic or mixed episodes in adults. These studies typically assessed symptom improvement using rating scales specific to bipolar disorder, such as the Young Mania Rating Scale (YMRS) or the Montgomery-Åsberg Depression Rating Scale (MADRS). Patients treated with iloperidone demonstrated significant reductions in manic symptoms, including elevated mood, increased energy, and impulsivity, leading to stabilization of mood and prevention of further escalation into manic episodes (Torres et al., 2024).

#### 4.10.5 Practical implementation issues

Iloperidone is administered orally twice daily without regard to meals, with dosage titration to avoid orthostatic hypotension. Dosage adjustments are recommended for patients with poor CYP2D6 metabolism or hepatic impairment. Monitoring includes baseline and ongoing complete blood counts, especially in patients with preexisting low white blood cell count or a history of leukopenia/neutropenia. Concomitant medication use and potential drug interactions should be considered (Vanda Pharmaceuticals Inc, 2009).

#### 4.10.6 Adverse effects

Common adverse reactions include dizziness, dry mouth, fatigue, nasal congestion, orthostatic hypotension, somnolence, tachycardia, and weight gain. Warnings highlight potential risks such as cerebrovascular adverse reactions, QT prolongation, neuroleptic malignant syndrome, metabolic changes, seizures, leukopenia, neutropenia, agranulocytosis, priapism, cognitive and motor impairment, and intraoperative floppy iris syndrome (Vanda Pharmaceuticals Inc, 2009).

### 4.11 Loxapine inhalation (Adasuve)

#### 4.11.1 Overview

Loxapine Inhalation (Adasuve) is an inhaled powder form of loxapine delivered through a novel device, offering a rapid alternative to intramuscular (IM) injections for managing acute agitation in patients with schizophrenia or bipolar I disorder. It acts by blocking postsynaptic mesolimbic D1 and D2 receptors, as well as serotonin 5-HT2 receptors, which contributes to its efficacy in symptom relief (Citrome, 2012; de Berardis et al., 2017) Adasuve can begin improving symptoms within just 10 min, with effects lasting up to 2 h. The maximum recommended dose is 10 mg within 24 h. Its use is restricted to select facilities equipped to manage its risks, as mandated by the FDA's Risk Evaluation and Mitigation Strategy (REMS), primarily due to a Black Box warning regarding the risk of bronchospasm that can lead to respiratory arrest.

#### 4.11.2 Indications

Acute agitation associated with schizophrenia or bipolar I disorder (2012).

#### 4.11.3 Dose and route

Inhalation powder 10 mg, once per day maximum.

#### 4.11.4 Evidence

Two studies of schizophrenia and bipolar disorder showed that loxapine inhalation of 5 mg and 10 mg were both superior to placebo, as early as 10 min, as evidenced by the PANSS Excited component scale. Pooled data from efficacy studies revealed the following effect sizes for inhaled loxapine: NNT = 4 for 5 mg vs. placebo (95% CI 3–5) and NNT = 3 for 10 mg vs. placebo (95% CI 3–4) (Citrome, 2011).

#### 4.11.5 Practical issues and implementation

Its use is restricted to approved facilities under the FDA-required REMS program due to the Black Box warning for bronchospasm risks. Higher cost as compared to oral or IM formulations.

#### 4.11.6 Adverse effects

Dysgeusia (14%), sedation (12%), respiratory distress in patients with asthma (54%), COPD (19%), and risk of bronchospasm.

### 4.12 Lumateperone Tosylate (Caplyta)

#### 4.12.1 Overview

Lumateperone is an orally administered dopamine and 5-HT2A receptor antagonist approved for schizophrenia and bipolar depression.

#### 4.12.2 Indications

• Schizophrenia in adults (2019).
• Bipolar I or II disorder: Depressive episodes as monotherapy (2021), and adjunctive therapy with lithium or valproate (2021).

#### 4.12.3 Dose and route

Oral 42 mg daily.

#### 4.12.4 Evidence

Clinical trials indicated significant efficacy in treating schizophrenia symptoms compared to placebo, with marked reductions in PANSS and CGI-S scores (Lieberman et al., 2016).

#### 4.12.5 Practical implementation issues

Because of its unique mechanism of action, lumateperone may offer effective treatment with fewer dopamine-related side effects.

#### 4.12.6 Adverse effects

• Somnolence, dry mouth, dizziness, and nausea.
• Risk of cerebrovascular events and metabolic changes.

### 4.13 Lurasidone (Latuda)

#### 4.13.1 Overview

Lurasidone, an orally administered D2 and serotonin 5-HT2A antagonist, is approved for treating schizophrenia in adults and adolescents, and for bipolar depression.

#### 4.13.2 Indications

Schizophrenia in adults (2010) and adolescents (13–17 years) (2017), Depressive episodes associated with bipolar I disorder (bipolar depression) in adults (2013) and pediatric patients (10–17 years) (2018) as monotherapy, and depressive episode associated with bipolar I disorder (bipolar depression) in adults as adjunctive therapy with lithium or valproate (2013).

#### 4.13.3 Dose and route

Oral 40–80 mg daily.

#### 4.13.4 Evidence

The Positive and Negative Syndrome Scale (PANSS) (primary outcome) in 6 weeks showed changes of −25.7, −23.6, and −16.0 in the 40 mg, 80 mg, and placebo groups, respectively (P < 0.05). The 15 mg olanzapine control group showed a change of −28.7. Changes in Clinical Global Impression showed changes of −1.5, −1.4, and −1.1 in the 40 mg, 120 mg, and placebo groups, respectively (P < 0.05). Changes in the 15 mg olanzapine group were −1.5 N = 478 (Stahl et al., 2013).

#### 4.13.5 Practical issues and implementation

Lurasidone is expensive, and no generic version is available. It is less likely to cause metabolic disturbance and excess serum prolactin levels (Javed et al., 2019).

#### 4.13.6 Adverse effects

Somnolence, nausea, and akathisia (Citrome et al., 2012).

### 4.14 Olanzapine and samidorphan (Lybalvi)

#### 4.14.1 Overview

Lybalvi is a novel formulation combining olanzapine, an antipsychotic, with samidorphan, an opioid antagonist, to treat schizophrenia and bipolar I disorder. This combination aims to mitigate the weight gain associated with olanzapine.

#### 4.14.2 Indications

• Schizophrenia (2021).
• Bipolar I disorder: Monotherapy for acute manic/mixed episodes (2021) and adjunctive therapy with lithium or valproate (2021).

#### 4.14.3 Dose and route

Oral 5 mg/10 mg–20 mg/10 mg.

#### 4.14.4 Evidence

Clinical trials evaluating Lybalvi from Day 8 to Day 92 showed no significant differences in PANSS scores between patients treated solely with olanzapine and those receiving combined therapy with varying doses of samidorphan (p = 0.3). However, the combined therapy demonstrated a statistically significant reduction in weight gain compared to olanzapine alone (p = 0.006) (Martin et al., 2019).

#### 4.14.5 Practical implementation issues

The medication is contraindicated with opioid use.

#### 4.14.6 Adverse effects

• Somnolence, weight gain, and metabolic changes (Citrome et al., 2021).
• Contraindicated in patients using opioids due to the risk of precipitated withdrawal.

### 4.15 Paliperidone extended release (Invega)

#### 4.15.1 Overview

Paliperidone Extended Release, an orally administered atypical antipsychotic, effectively blocks D2, 5-HT2A, alpha-1, alpha-2, and H1 receptors. It is uniquely FDA-approved to treat schizoaffective disorder.

#### 4.15.2 Indications

• Acute treatment of schizoaffective disorder as monotherapy (2009)
• Acute treatment of schizoaffective disorder as an adjunct to mood stabilizers and/or antidepressants (2009)
• Acute and maintenance treatment of schizophrenia (2006).

#### 4.15.3 Dose and route

Oral 3–12 mg daily.

#### 4.15.4 Evidence

In a 6-week randomized controlled trial, paliperidone extended-release (ER) tablets were assessed for their efficacy in treating acute schizophrenia. The study demonstrated that both 6 mg and 12 mg doses of paliperidone ER significantly improved PANSS total scores compared to placebo, with the 6 mg dose also showing a significant improvement in personal and social performance. The 6 mg dose had a similar incidence of adverse events as placebo, with a slight increase observed for the 12 mg dose. The study concludes that paliperidone ER, especially at the 6 mg dose, is an effective treatment for schizophrenia, offering both improved symptoms and good tolerability (Marder et al., 2007).

#### 4.15.5 Practical issues and implementation

Long-acting injectable (LAI) forms of paliperidone are available in monthly, bimonthly, and twice-yearly forms.

#### 4.15.6 Adverse effects

• Extrapyramidal symptoms, tachycardia, akathisia, and somnolence.

### 4.16 Cobenfy (Xanomeline plus Trospium)

#### 4.16.1 Overview

Cobenfy combines xanomeline, a muscarinic M1/M4 agonist, with trospium chloride, a non-centrally acting muscarinic antagonist. This oral formulation was approved by the FDA in October 2024 for the treatment of schizophrenia in adults and is also being explored for Alzheimer's disease. It is the first antipsychotic to target cholinergic receptors rather than the traditional dopamine receptors.

#### 4.16.2 Indications

• Schizophrenia treatment in adults (2024).

#### 4.16.3 Dose and route

Oral, starting at 50 mg xanomeline/20 mg trospium twice daily, up to 125 mg/30 mg twice daily.

#### 4.16.4 Evidence

In Phase 3 trials, xanomeline significantly improved psychotic symptoms and cognitive functions such as working memory and linguistic cognition in patients with schizophrenia, as measured by changes in the Brief Psychiatric Rating Scale (BPRS) and PANSS scale (Shekhar et al., 2008).

#### 4.16.5 Adverse effects

• The most common side effects (>5%) included: constipation, dyspepsia, nausea, vomiting, headache, increases in blood pressure, dizziness, acid reflux, abdominal discomfort, and diarrhea (Kaul et al., 2024).
• Phase 3 reported similar discontinuation rates between Cobenfy (7%) and placebo (6%), and similar rates to placebo (2%) in serious side effects such as suicidal ideation, worsening of schizophrenia symptoms, and appendicitis.
• Limited anticholinergic adverse events were reported.

## 5 Detailed descriptions of pipeline medications in PHASE-3 for schizophrenia as of December 31, 2024 (summarized in Table 4) except for an LAI which is fully detailed in a separate manuscript on LAIs

### 5.1 Brilaroxazine (Oxaripiprazole; RP-5063)

#### 5.1.1 Overview

Brilaroxazine, related structurally to aripiprazole and brexpiprazole, is an orally administered partial agonist at dopamine D2, D3, and D4 receptors, as well as serotonin 5-HT1A, 5-HT2A, 5-HT2B receptors. It exhibits antagonistic effects on serotonin 5-HT6 and 5-HT7 receptors. Demonstrating superiority over placebo in prior Phase 2 trials, it is currently in Phase 3 focusing on cognitive dysfunction and psychotic symptoms in schizophrenia.

#### 5.1.2 Monotherapy or Add-on

Monotherapy**.**

#### 5.1.3 Preliminary findings

Brilaroxazine is being developed as a monotherapy for schizophrenia, with clinical trials focusing on its standalone efficacy and safety. Phase II trials demonstrated significant improvements in both positive and negative symptoms, and preliminary data from ongoing Phase III trials further support its favorable safety profile and efficacy. Notably, no adjunctive treatment arms were included in these studies, underscoring its development as a primary treatment option for schizophrenia (Saha et al., 2005).

#### 5.1.4 Dose and route

Oral, 5–100 mg daily.

#### 5.1.5 Evidence

In animal models, brilaroxazine showed a significant reduction in schizophrenia-associated cognitive impairment and exhibited notable antipsychotic activity following acute administration (Rajagopal et al., 2017).

#### 5.1.6 Practical implementation issues

Administered orally, brilaroxazine may pose risks such as orthostatic hypotension, nausea, and dizziness.

#### 5.1.7 Adverse effects

• Reported side effects include orthostatic hypotension, nausea, and dizziness.

### 5.2 Iclepertin (BI-425809)

#### 5.2.1 Overview

Iclepertin, an orally administered glycine transporter-1 (GlyT1) inhibitor, has been granted Breakthrough Therapy Designation by the FDA. It is under Phase 3 investigation for cognitive dysfunction in schizophrenia.

#### 5.2.2 Preliminary findings

Improvements in cognitive function in schizophrenia patients.

#### 5.2.3 Dose and route

Oral, 2–25 mg daily.

#### 5.2.4 Monotherapy or Add-on

Add-on therapy.

#### 5.2.5 Evidence

Iclepertin significantly improved cognitive function in schizophrenia patients compared to placebo, as evidenced by enhancements in MATRICS Consensus Cognitive Battery (MCCB) scores. Phase 1 trials reported enhanced functioning and reversal of cognitive impairment due to increased glycine levels in plasma and cerebrospinal fluids. Phase 2 trials indicated that doses of 2 mg–25 mg improved cognitive processing over 12 weeks, with notable changes in MCCB scores (Rosenbrock et al., 2023).

The ongoing Phase III CONNEX trials are specifically designed to evaluate the efficacy of Iclepertin as an adjunctive treatment for cognitive impairment in patients with schizophrenia who are already receiving stable doses of antipsychotic medications. These trials focus on cognitive endpoints and aim to confirm the initial positive safety and efficacy findings from earlier studies. Initial results are promising, showing significant cognitive improvements with a good safety profile (Rosenbrock et al., 2023).

#### 5.2.6 Adverse effects

• In Phase 2 trials, adverse effects were reported in up to 59% of participants at various doses, with gastrointestinal side effects being the most prevalent (Fleischhacker et al., 2021).
• Phase 3 data on adverse effects is pending.

### 5.3 Raloxifene (as an adjunct)

#### 5.3.1 Overview

Raloxifene, an estrogen receptor modulator, was tested as an adjunctive therapy in three Phase 3 trials focusing on positive, negative, and cognitive symptoms of schizophrenia. One Phase 3 trial showed potential benefit for positive and negative symptoms of schizophrenia in postmenopausal women, while another showed possible benefit in cognitive deficits in women with schizophrenia.

#### 5.3.2 Dose and route

Oral, 120 mg daily.

#### 5.3.3 Monotherapy or Add-on

Add-on therpay

#### 5.3.4 Preliminary findings and evidence

Raloxifene is primarily studied as an adjunctive treatment for schizophrenia, especially in postmenopausal women. Kulkarni et al. demonstrated that adjunctive raloxifene (120 mg/day) significantly reduced illness severity (PANSS total score) in refractory schizophrenia compared to placebo, highlighting its efficacy with antipsychotics (Kulkarni et al., 2016). Similarly, Weickert et al. found that adjunctive raloxifene improved attention and memory without significantly reducing symptom severity, suggesting benefits for cognitive deficits (Weickert et al., 2015). Usall et al. reported that adjunctive raloxifene (60 mg/day) significantly reduced negative, general, and total symptoms in postmenopausal women, corroborating earlier findings by the same group (Usall et al., 2011, 2016). Additional studies, including a meta-analysis by Zhu et al., confirmed improved psychotic symptoms in postmenopausal women, while Ji et al. demonstrated enhanced neural activity linked to social cognition with raloxifene (Ji et al., 2016; Zhu et al., 2018). In summary, raloxifene is intended as an adjunctive therapy for schizophrenia, particularly in postmenopausal women, with consistent evidence supporting its efficacy in reducing symptoms and improving cognitive function.

#### 5.3.5 Adverse effects

• Hot flashes (29%), flu-like symptoms, and sinusitis.
• Increased risk of deep vein thrombosis and pulmonary embolism have been detected.

### 5.4 Roluperidone (MIN-101)

#### 5.4.1 Overview

Roluperidone is an orally administered antagonist at serotonin 5-HT2A and alpha 1-a adrenergic receptors. It has completed a Phase 3 trial and received a New Drug Application for treating negative symptoms in schizophrenia patients.

#### 5.4.2 Dose and route

Oral, 32–64 mg daily.

#### 5.4.3 Monotherapy or Add-on

Monotherapy.

#### 5.4.4 Preliminary findings and evidence

Roluperidone (MIN-101) is a novel antipsychotic targeting 5-HT2A, sigma2, and α1A-adrenergic receptors, developed specifically for negative symptoms of schizophrenia. Clinical trials have demonstrated its efficacy and safety as a monotherapy. A placebo-controlled trial showed significant improvements in the PANSS-derived Negative Symptom Factor Score (NSFS) and Personal and Social Performance (PSP) score, highlighting its potential to reduce negative symptoms and enhance daily functioning (Davidson et al., 2022).

Long-term studies (24–40 weeks) confirmed sustained benefits in negative symptoms and social functioning with good tolerability and low rates (<10%) of symptomatic worsening. No significant changes in vital signs, weight, metabolic indices, or extrapyramidal symptoms were observed (Rabinowitz et al., 2023). Network analysis indicates that Roluperidone primarily targets avolition, central to negative symptom reduction, as evidenced in phase 2b and 3 trials (James et al., 2024; Strauss et al., 2020). In summary, Roluperidone is a promising monotherapy for negative symptoms of schizophrenia, with robust evidence from short- and long-term trials supporting its efficacy and safety profile.

#### 5.4.5 Adverse effects

• Worsening of schizophrenia (8.0% vs. 3.0% placebo), anxiety (4.0% vs. 2.0% placebo), and agitation (3.0% vs. 2.0% placebo).
• Discontinuation due to side effects occurred in 5% of patients on placebo, 9% of patients on 64 mg, and 11% of patients on 32 mg (Davidson et al., 2022).

### 5.5 Sodium benzoate (Naben)

#### 5.5.1 Overview

Sodium benzoate, traditionally a food preservative, is being explored as a treatment for schizophrenia. It inhibits D-amino acid oxidase (DAAO), which may enhance NMDA receptor function by preventing the degradation of D-serine, a co-agonist of these receptors. This mechanism aims to mitigate NMDA receptor hypofunction implicated in schizophrenia. Adjunctive treatment for schizophrenia and clozapine-resistant schizophrenia (Seetharam et al., 2022; Tsapakis et al., 2023).

#### 5.5.2 Dose and route

Oral, 500–1000 mg twice a day.

#### 5.5.3 Monotherapy or Add-on

Add-on therapy.

#### 5.5.4 Preliminary findings and evidence

Lin et al. demonstrated that sodium benzoate (1–2 g/day) significantly improved negative symptoms and overall symptomatology in clozapine-resistant schizophrenia, with better PANSS and Quality of Life scores compared to placebo (Lin et al., 2018). Lane et al. reported similar findings, with large effect sizes across symptom domains and cognitive improvements in chronic schizophrenia (Lane et al., 2013). Baker et al. outlined a dose-finding trial to optimize sodium benzoate's efficacy and explore its mechanisms, focusing on PANSS, GAF, and CGI measures (Baker et al., 2021). However, Scott et al. found no significant benefits in early psychosis, suggesting its effectiveness is limited to chronic or treatment-resistant conditions (Scott et al., 2020). In summary, sodium benzoate is an effective adjunctive treatment for schizophrenia, particularly in chronic or clozapine-resistant cases, improving symptoms and cognitive function when combined with standard antipsychotics.

#### 5.5.5 Adverse effects

While initial studies showed a generally good safety profile, a subsequent meta-analysis indicated significantly higher extrapyramidal symptoms, highlighting the need for careful monitoring of motor side effects during long-term use (Strauss et al., 2020).

### 5.6 Ulotaront (SEP-363856)

#### 5.6.1 Overview

Ulotaront acts as an agonist at trace amine-associated receptor-1 (TAAR-1) and serotonin 5-HT1A receptors. It has been granted Breakthrough Therapy designation by the FDA and is currently in Phase 3 trials for psychotic symptoms of schizophrenia.

#### 5.6.2 Dose and route

Oral, 25–75 mg daily.

#### 5.6.3 Monotherapy or Add-on

Ulotaront was intended as a monotherapy for schizophrenia without the need for adjunctive treatments.

#### 5.6.4 Preliminary findings and evidence

In a double-blind, placebo-controlled Phase II trial, Ulotaront significantly improved the Positive and Negative Syndrome Scale (PANSS) total score compared to placebo, with an effect size of 0.45. Post-hoc analyses further supported its efficacy in reducing negative symptoms. The open-label extension study confirmed sustained improvements across schizophrenia symptoms and a favorable safety profile, with a 6-month completion rate of 67% (Achtyes et al., 2023).

Unfortunately, the Phase 3 clinical trials of Ulotaront did not achieve their primary endpoints. In July 2023, Sumitomo Pharma and Otsuka Pharmaceutical announced that the DIAMOND 1 and DIAMOND 2 studies, which evaluated Ulotaront in adults with acute schizophrenia, failed to meet their primary goals. The companies suggested that an unusually high placebo response might have obscured the drug's therapeutic effects, a phenomenon documented in psychiatric studies (Pharma and Otsuka Pharmaceutical, 2023).

#### 5.6.5 Adverse effects

• Common adverse effects (>5% of participants) included headaches, insomnia, and anxiety.
• Serious adverse effects of concern included psychosis, depression, and possible suicidal ideation.
• Phase 3 trials are ongoing, with body weight being a consideration for proper dosage administration (Galluppi et al., 2021).

### 5.7 Vortioxetine (Trintellix)

#### 5.7.1 Overview

Vortioxetine, an atypical antidepressant approved by the FDA in 2013 for MDD, possesses a multifaceted mechanism of action. It inhibits the serotonin transporter while modulating various serotonin receptors, thereby enhancing the release of other neurotransmitters such as acetylcholine, norepinephrine, histamine, and dopamine. Beyond its antidepressant effects, vortioxetine is now being explored as a potential adjunctive therapy for schizophrenia treatment, particularly to address cognitive and negative symptoms.

#### 5.7.2 Dose and route

Oral, 5–20 mg daily.

#### 5.7.3 Monotherapy or Add-on

It has been investigated for its potential benefits in treating schizophrenia, particularly as an adjunctive treatment.

#### 5.7.4 Preliminary findings

Vortioxetine has demonstrated potential therapeutic effects when used as an adjunctive therapy to antipsychotics in patients with schizophrenia, particularly those with significant negative symptoms.

#### 5.7.5 Evidence

A randomized, double-blind, placebo-controlled trial by Moazen-Zadeh et al. demonstrated that vortioxetine, when added to risperidone, significantly improved negative symptoms and overall symptomatology in patients with chronic schizophrenia. The study found that adjunctive vortioxetine (10 mg b.i.d.) was well tolerated and produced better improvements than placebo in the Positive and Negative Syndrome Scale (PANSS) total score and Quality of Life Scale (Moazen-Zadeh et al., 2020).

Further supporting its adjunctive use, a pilot study by Bruno et al. outlined that vortioxetine supplementation significantly improved cognitive function in patients on stable clozapine treatment. The study reported significant improvements in the Stroop test and Semantic Fluency, as well as reductions in PANSS positive and total scores and depressive symptoms (Bruno et al., 2020).

Preclinical studies have also shown promising results. Bozkurt and Unal demonstrated that vortioxetine improved negative and cognitive symptoms in a subchronic MK-801 model of schizophrenia in rats. The study suggested potential therapeutic effects on behavioral and molecular deficits. Additionally, Taskiran et al. found that vortioxetine improved schizophrenia-like behavioral deficits in a Poly I:C-induced maternal immune activation model of schizophrenia in rats, indicating its potential benefits on negative and cognitive symptoms (Bozkurt and Unal, 2023; Taskiran et al., 2024).

In summary, vortioxetine is intended for use as an adjunct to existing antipsychotic treatments for schizophrenia, particularly targeting negative and cognitive symptoms. The evidence consistently supports its efficacy in improving symptom severity and cognitive function when used in combination with standard antipsychotic medications.

#### 5.7.6 Adverse effects

• Common adverse effects (>5% of participants) included nausea, constipation, and vomiting.
• Risk of activation of mania, bleeding, fragility fractures, hyponatremia, serotonin syndrome, sexual dysfunction, suicidal thinking/behavior, and withdrawal syndrome.
• Might require dose adjustment with CYP2D6 inducers (e.g., carbamazepine, phenytoin, barbiturates) or inhibitors (e.g., bupropion, fluoxetine, paroxetine) (Moazen-Zadeh et al., 2020).

## 6 Discussion

From 2008 to December 31, 2024, there are 29 available medications for schizophrenia (of which 13 are long-acting injectables). The 13 LAIs approved from 2008 to December 31, 2024, are fully detailed by the authors in a separate manuscript (Meyer et al., 2024). As of December 31, 2024, 8 pipeline medications for schizophrenia are currently in Phase 3 clinical trials, including one LAI, which are all fully detailed in a separate manuscript.

In addition to these 29 medications, several antipsychotic medications widely used in Europe for the treatment of schizophrenia remain unavailable in the United States, despite their established efficacy and favorable safety profiles. Amisulpride, zotepine, and sertindole have each demonstrated significant therapeutic benefits, particularly in addressing negative symptoms and reducing extrapyramidal side effects (EPS) yet have not been integrated into U.S. practice.

Amisulpride, a dopamine D2/D3 antagonist with limbic selectivity, is noted for its efficacy in treating negative symptoms and its low EPS profile (Leucht et al., 2013; Huhn et al., 2019; Wetzel et al., 1998; Mota et al., 2002; Isbister et al., 2010). Similarly, zotepine, approved in Germany and Japan, offers an alternative to clozapine for treatment-resistant schizophrenia, with significant improvements in both positive and negative symptoms and minimal EPS (DeSilva et al., 2006; Lin et al., 2013; Palmgren et al., 2000). Lastly, sertindole combines efficacy in addressing negative symptoms with a favorable metabolic and EPS profile, although concerns about QT prolongation led to its withdrawal and subsequent reintroduction under strict monitoring (Juruena et al., 2011; Lewis et al., 2000; Cincotta and Rodefer, 2010; Pae, 2013).

The limited availability of these medications in the U.S. represents a gap in the therapeutic landscape for schizophrenia. Addressing the regulatory and clinical barriers to their adoption could enhance treatment options and provide meaningful benefits for patients with unmet clinical needs.

### 6.1 Progress in schizophrenia treatment

Over the past decade, schizophrenia treatment has seen significant advancements, particularly with the approval of new medications and formulations aimed at improving symptom management, adherence, and patient quality of life. While the increasing number of therapies in the pipeline suggests progress, it remains critical to assess whether these innovations truly address the pressing unmet needs in schizophrenia care or simply reflect incremental improvements over existing treatments.

### 6.2 Addressing cognitive and negative symptoms

Schizophrenia is a complex, multifaceted disorder, encompassing cognitive deficits, negative symptoms, and psychosis, all of which contribute to poor functional outcomes and reduced quality of life. Medications like brilaroxazine and iclepertin have been specifically developed to target the cognitive and negative symptoms of schizophrenia—areas that are historically resistant to treatment with conventional antipsychotics. Brilaroxazine, a dual dopamine-serotonin modulator, has shown promise in enhancing cognitive function, while iclepertin, a GlyT1 inhibitor, works by enhancing NMDA receptor activity, potentially improving cognitive deficits. Despite their promise, the key question remains whether these therapies represent true breakthroughs or simply refined iterations of previous drug classes.

Similarly, negative symptoms, such as social withdrawal, apathy, and emotional blunting, continue to be inadequately addressed by conventional antipsychotics. New medications, including roluperidone and raloxifene, target these symptoms more directly, with roluperidone affecting serotonin and adrenergic receptors, and raloxifene—traditionally used for osteoporosis—being explored for its potential to address both cognitive and negative symptoms. However, it is essential to critically evaluate whether these drugs offer genuinely novel benefits or are simply incremental steps forward within a familiar pharmacological framework.

### 6.3 Novel drug classes and mechanisms of action

The emergence of novel drug classes presents additional opportunities for enhanced efficacy and fewer side effects. Ulotaront, a TAAR-1 agonist, represents a completely new class of antipsychotic drugs that offer the potential for more effective symptom control and fewer side effects compared to traditional dopamine antagonists. Similarly, sodium benzoate, a compound traditionally used as a food preservative, is being investigated for its ability to enhance NMDA receptor function, offering hope for those with clozapine-resistant schizophrenia. These novel approaches provide valuable alternatives, but their long-term safety, efficacy, and comparative advantage over established therapies remain subjects for further research.

### 6.4 Improving adherence with digital health innovations

One critical trend in current schizophrenia management is the development of formulations designed to improve medication adherence. Adherence remains a major challenge in schizophrenia treatment, with nonadherence often leading to relapse and rehospitalization. Abilify MyCite, an aripiprazole formulation embedded with a digital sensor, exemplifies the integration of digital health technologies into schizophrenia treatment. This system allows real-time monitoring of medication adherence, potentially enhancing treatment outcomes by providing clinicians with valuable data to guide clinical decisions. While the potential benefits are clear, barriers such as cost, patient engagement, and privacy concerns must be addressed for the widespread adoption of such digital tools. If successfully integrated, they could pave the way for more personalized, data-driven care that enhances both the clinical management and patient experience.

### 6.5 Advances in safety and side effect profiles

Furthermore, several newer antipsychotic medications, such as lumateperone and lurasidone, have emerged with favorable side effect profiles, particularly in terms of reducing metabolic disturbances like weight gain and hyperglycemia—issues that significantly impact patient quality of life and adherence. These drugs provide an alternative to older agents known for their adverse effects, offering the potential for better long-term outcomes. However, their incorporation into treatment regimens requires careful attention to both their efficacy in symptom management and their cost-effectiveness, ensuring they provide tangible improvements in real-world clinical practice (Kantrowitz et al., 2023).

### 6.6 LAIs in schizophrenia management

The Phase 3 pipeline for schizophrenia features a number of innovative agents, including long-acting injectables (LAIs) such as aripiprazole, olanzapine, paliperidone, and risperidone. LAIs have proven particularly beneficial for patients who struggle with daily oral medication adherence, providing a sustained-release option to maintain therapeutic drug levels. The ability to manage treatment adherence more effectively with LAIs adds an important tool to the schizophrenia treatment landscape.

### 6.7 Expanding the treatment paradigm

In addition to these developments, the current landscape of novel mechanisms of action includes TAAR-1 agonism, muscarinic receptor agonism, serotonin receptor antagonism/inverse agonism, and glutamatergic modulation, offering targeted treatments for cognitive and negative symptoms of schizophrenia. These new agents may offer significant advantages over traditional dopamine antagonists by addressing some of the most challenging aspects of the disease, such as cognitive impairment and treatment resistance. However, these drugs are still undergoing trials, and their long-term benefits and safety profiles need further investigation to establish their place in the treatment paradigm.

### 6.8 The future: personalized medicine in schizophrenia care

Looking to the future, personalized medicine holds great promise for optimizing schizophrenia treatment. Advances in genetic profiling and biomarker discovery could enable clinicians to select medications with greater precision, reducing trial-and-error prescribing and improving patient outcomes. The integration of personalized medicine with digital health technologies could further revolutionize care by providing real-time insights into treatment efficacy, allowing clinicians to adjust regimens dynamically based on patient responses. This combination of pharmacological and technological innovation could lead to more tailored, individualized treatment plans, significantly improving long-term outcomes for patients with schizophrenia.

### 6.9 Innovation or incremental progress?

While the current pipeline of medications represents exciting potential, it is essential to critically assess whether these new treatments provide true breakthroughs or merely incremental advancements. The ultimate goal is to address the core unmet needs of schizophrenia, including cognitive deficits, negative symptoms, and medication adherence. As the field continues to evolve, the integration of these new therapies with more personalized and data-driven approaches could transform the way schizophrenia is treated, improving both the clinical management and quality of life for patients.

## 7 Conclusion

This review encapsulates a dynamic era in the treatment of schizophrenia from 2008 to 2024, characterized by the approval of 29 medications for schizophrenia including 13 long-acting injectables. The emergence of long-acting injectables and Phase 3 pipeline medications indicate a pivotal shift toward more tailored and potentially more effective treatment modalities. These developments not only promise to enhance patient care and quality of life but also signify major progress in personalizing the management of schizophrenia. Continued research and the clinical application of these findings will be vital in realizing the full potential of these therapeutic advances.

## Authors' Contributions Statement

Salma Abdelmoteleb, MD: Writing – original draft, reviewing & editing, Data curation, Formal analysis. Jayant Totlani, DO candidate: Writing – original draft, Data curation, Formal analysis. Salma Ramadan, MD: Writing – reviewing & editing, Methodology, Data curation. Mohamed Salem, BS: Data acquisition, Writing – reviewing & editing, Formal analysis. Ashley Meyer, MD candidate: Writing – reviewing & editing, Data curation. Tiffany Chang, PharmD candidate: Writing – reviewing & editing, Data curation. Madeline Ewing, Volunteer: Data acquisition, Writing – reviewing & editing. Luiza Freire, MD: Writing – reviewing & editing, Formal analysis. Nathalie Murphy, MD: Writing – reviewing & editing, Data acquisition. Sabrina Renteria, MD: Writing – reviewing & editing, Data acquisition. Romana Dymkoski, DO: Writing – reviewing & editing, Methodology. Omer Liran, MD: Writing – reviewing & editing, Supervision, Conceptualization. Rebecca Hedrick, MD: Writing – reviewing & editing, Formal analysis. Itai Danovitch, MD: Writing – reviewing & editing, methodology. Robert N. Pechnick, PhD: Writing – reviewing & editing, Supervision, Formal analysis. Waguih William IsHak, MD: Conceptualization, Supervision, Writing – reviewing & editing

## Funding

None.

## Conflict of interest

All authors declare that there are no actual or potential conflicts of interest, including financial, personal, or other relationships with individuals or organizations within the past three years, that could inappropriately influence, or be perceived to influence, the work submitted.
